# Machine Learning for IoT Applications and Digital Twins

**DOI:** 10.3390/s24155062

**Published:** 2024-08-05

**Authors:** Javad Rezazadeh, Omid Ameri Sianaki, Reza Farahbakhsh

**Affiliations:** 1 Crown Institute of Higher Education (CIHE), Sydney, NSW 2060, Australia; 2 Victoria University Business School, Victoria University, Melbourne, VIC 8001, Australia; omid.amerisianaki@vu.edu.au; 3 Institute Polytechnique de Paris, Telecom SudParis, 91000 Evry, France; reza.farahbakhsh@it-sudparis.eu

The Internet of Things (IoT) stands as one of the most transformative technologies of our era, significantly enhancing the living conditions and operational efficiencies across various domains. By interconnecting a multitude of sensors embedded within devices and machines, IoT generates vast amounts of data, commonly referred to as Big Data. This surge in data necessitates the development of advanced tools and techniques capable of processing, analyzing, and transforming these data into actionable knowledge [1]. The essence of IoT lies in its ability to capture real-time data from diverse sources, creating a complex network of information flow [2]. However, the sheer volume and velocity of these data present considerable challenges in terms of storage, processing, and the extraction of meaningful insights. To address these challenges, machine learning (ML) has emerged as a critical component in the IoT ecosystem [3]. Machine learning algorithms are extensively applied to IoT sensor data to achieve predictions, classifications, data associations, and conceptualizations. These algorithms enable the extraction of valuable information that can inform decision-making processes, optimize operations, and enhance user experiences [4]. Parallel to the advancements in IoT and machine learning is the concept of the digital twin. A digital twin is a sophisticated integration of IoT, artificial intelligence (AI), and software analytics, creating a virtual replica of physical entities. This digital counterpart can simulate, predict, and optimize real-world operations, providing unprecedented insights and control. The synergy between digital twin technology and IoT fosters the creation of dynamic, data-driven environments that can adapt and respond to changing conditions in real time [5].

This Special Issue invites original contributions and review papers that explore these themes, aiming to advance the knowledge and practical implementation of machine learning for IoT applications and digital twin technologies. The integration of these cutting-edge technologies promises to drive significant advancements across numerous fields, ultimately leading to smarter, more efficient, and more responsive systems.

The rapid evolution of technology has brought forth an era where the convergence of machine learning (ML), Internet of Things (IoT), and digital twins (DTs) is transforming industries and everyday life. The collection of research presented in this Special Issue highlights the cutting-edge advancements and innovative applications of these technologies across various fields, addressing some of the most pressing challenges and opening up new avenues for exploration and development.

The first paper, “A Machine Learning Approach as a Surrogate for a Finite Element Analysis: Status of Research and Application to One Dimensional Systems” [6], showcases the integration of ML algorithms with finite element analysis (FEA) to enhance maintenance scheduling for mechanical systems. By accurately predicting stress distributions using real-time data, this approach promises to improve safety and efficiency in maintenance procedures, illustrating the potential of ML in structural health monitoring.

In “A Smart Biometric Identity Management Framework for Personalized IoT and Cloud Computing-Based Healthcare Services” [7], the authors present a novel framework that leverages multimodal biometric traits and homomorphic encryption (HE) to ensure secure and reliable authentication in healthcare systems. This framework not only enhances security but also ensures the privacy of patients’ data, demonstrating the critical role of ML in advancing personalized healthcare services.

The study “Intelligent Tensioning Method for Prestressed Cables Based on Digital Twins and Artificial Intelligence” [8] addresses the complexities of cable tensioning in prestressed steel structures. By integrating DTs and AI, the proposed method offers intelligent control and safety evaluation, showcasing the synergy between these technologies in enhancing structural integrity and safety.

“A Framework for an Indoor Safety Management System Based on Digital Twin” [9] explores the application of DTs in improving indoor safety management. Utilizing IoT, building information modeling (BIM), and support vector machines (SVMs), this framework provides a comprehensive solution for monitoring and managing indoor safety, exemplifying the use of advanced technologies in smart building management.

In “A Generalized Threat Model for Visual Sensor Networks” [10], the authors tackle the security challenges in visual sensor networks (VSNs). By presenting a threat model and classifying vulnerabilities, this research work highlights the importance of robust security measures in ensuring the reliability of VSNs in various smart environments.

The paper “Covert Timing Channel Analysis Either as Cyber Attacks or Confidential Applications” [11] investigates the behavior of covert timing channels in IoT networks. By analyzing packet delay thresholds, this study offers insights into detecting and mitigating security threats, emphasizing the need for advanced security protocols in IoT systems.

“Determining the Optimal Restricted Driving Zone Using Genetic Algorithm in a Smart City” [12] addresses traffic control in metropolitan areas. Using a genetic algorithm to optimize restricted traffic zones, this research work aims to balance traffic congestion and citizen satisfaction, showcasing the potential of ML in urban planning and management.

In “Federated Reinforcement Learning for Training Control Policies on Multiple IoT Devices” [13], the authors propose a federated reinforcement learning architecture to enhance the learning process across multiple IoT devices. This collaborative approach accelerates learning and improves control policies, demonstrating the benefits of federated learning in IoT applications.

Finally, “Digital Twin Coaching for Physical Activities: A Survey” [14] provides a comprehensive review of digital twin technology in the context of physical activity coaching. Highlighting current research, interactivity, and privacy concerns, this survey sets the stage for future developments in digital coaching, underlining the significance of DTs in promoting health and wellness.

This collection of papers represents the forefront of research in machine learning, IoT, and digital twins, showcasing their transformative potential across various domains. As you delve into these studies, we hope that you find inspiration and insights that drive further innovation and progress in this exciting and dynamic field.
